# Phenobarbital versus morphine in the management of neonatal abstinence syndrome, a randomized control trial

**DOI:** 10.1186/s12887-015-0377-9

**Published:** 2015-05-15

**Authors:** Fatemeh Nayeri, Mahdi Sheikh, Majid Kalani, Pedram Niknafs, Mamak Shariat, Hosein Dalili, Ahmad-Reza Dehpour

**Affiliations:** Maternal, Fetal and Neonatal Research Center, Tehran University of Medical Sciences, Tehran, Iran; Breastfeeding Research Center, Tehran University of Medical Sciences, Tehran, Iran; Department of Neonatology, Akbar-Abadi Hospital, Iran University of Medical Sciences, Tehran, Iran; Pediatrics Department, Kerman University of Medical Sciences, Kerman, Iran; Department of Pharmacology, Faculty of Medicine, Tehran University of Medical Sciences, Tehran, Iran

**Keywords:** Addicted, Dependency, Opioid, Pregnancy, Withdrawal

## Abstract

**Backgrounds:**

Evaluating the efficacy of the loading and tapering dose of Phenobarbital versus oral Morphine in the management of NAS.

**Methods:**

This randomized, open-label, controlled trial was conducted on 60 neonates born to illicit drugs dependent mothers at Vali-Asr and Akbar-Abadi hospitals, Tehran, Iran, who exhibited NAS requiring medical therapy. The neonates were randomized to receive either: Oral Morphine Sulfate or a loading dose of Phenobarbital followed by a tapering dose. The duration of treatment required for NAS resolution, the total hospital stay and the requirement for additional second line treatment were compared between the treatment groups.

**Results:**

The Mean ± Standard Deviation for the duration of treatment required for the resolution of NAS was 8.5 ± 5 days in the Morphine group and 8.5 ± 4 days in the Phenobarbital group (*P* = 0.9). The duration of total hospital stay was 12.6 ± 5.6 days in the Morphine group and 12.5 ± 5.3 days in the Phenobarbital group (*P* = 0.7). 3.3 % in the Morphine group versus 6.6 % in the Phenobarbital group required adjunctive treatment (*P* = 0.5).

**Conclusions:**

There were no significant differences in the duration of treatment, duration of hospital stay, and the requirement for adjunctive treatment, between the neonates with NAS who received Morphine Sulfate and neonates who received a loading and tapering dose of Phenobarbital.

**Trial registration:**

This study is registered at the Iranian Registry of Clinical Trials (www.irct.ir) which is a Primary Registry in the WHO Registry Network. (Registration Number = IRCT201406239568N8)

## Background

Neonatal Abstinence Syndrome (NAS) is a set of signs and symptoms that can occur in the neonates due to intrauterine exposure to the opioids consumed by mothers. NAS is characterized by gastrointestinal, central nervous system, autonomic and respiratory signs and symptoms [1]. Although the syndrome mostly occurs in the context of antepartum opioid use, with the growing prevalence of illicit drug use, other drugs have also been described to cause NAS [2–7].

Based on a recent National Survey on Drug Use and Health (NSDUH) in the United States 5.9 % of pregnant women reported using illicit drugs during gestation [8]. Among the neonates who were exposed to illicit drugs in utero, withdrawal signs and symptoms requiring medical intervention develop in 27–91 % [4]. The treatment rate is usually higher among poly substance and opioid exposed neonates (60–70 %) and lower in stimulants only exposed neonates (less than 10 %) whose the majority do not require pharmacologic treatment [4–7, 9].

In the last decade with the increase in the incidence of NAS, the total hospital charges have dramatically increased [2].

Prompt diagnosis and treatment of NAS is important because of its serious and potentially life threatening complications [10]. Although non-pharmacologic treatments have been proposed for the management of NAS, especially for the neonates born to cocaine or methamphetamines only dependent mothers [5], most neonates with NAS require pharmacologic treatment [4, 11]. Many agents have been proposed for the treatment of NAS including opioids, anticonvulsants, α2-adrenergic antagonists, benzodiazepines, chloral hydrate and chlorpromazine [11]. However the optimal pharmacologic treatment of NAS has not been established and is still debated [3, 11]. In addition despite all the proposed treatments the duration of hospital stay for NAS remained relatively unchanged during the past decade [2].

Morphine is the most commonly used drug as an initial therapy for the treatment of NAS, however the evidence suggesting the effect of prolonged opioid exposure on the developing neonatal brain made the researchers search for an alternative drug for initial NAS treatment [12]. Phenobarbital is another drug widely used for the treatment of non-opioid NAS [4, 13]. some studies tried to compare Phenobarbital versus Morphine in the treatment of NAS; In the studies of Jackson et al. and Ebner et al. shorter treatment duration was noted in the Morphine treated group compared to the Phenobarbital treated group [14, 15].

In the study of Jackson et al. all the mothers had received Methadone during pregnancy. Also in the study of Ebner et al. the mothers were only opioid dependent. Considering the increasing prevalence of polysubstance use during pregnancy [16], and that Phenobarbital is preferred in the NAS due to intrauterine polysubstance exposure [4, 13], studies are needed to compare Phenobarbital versus Morphine treatment in the neonates born to mothers who used other addictive drugs in addition to opioids during gestation. Additionally it is documented that the use of loading dose of Phenobarbital significantly reduces the time for symptom control in neonates with NAS [17]. In the available studies no loading dose was used, therefore it is still unknown if there is any differences in the duration of treatment and hospital stay in the Morphine versus Phenobarbital treated groups when loading dose of Phenobarbital is used [18].

We conducted this study to compare the efficacy of initial Phenobarbital treatment with loading and maintenance dose versus Morphine treatment in the neonates born to drug (opioids, stimulants and polysubstance) dependent mothers who exhibit NAS requiring pharmacologic treatment. The primary goal of the study was to evaluate the duration of treatment required for NAS resolution. The total hospital stay and the requirement for additional second line treatment were secondary aims of the study.

## Methods

### Study population and study design

This multicentric prospective, randomized, open label, trial was conducted on 60 neonates who were born to illicit drugs dependent mothers and exhibited NAS requiring medical therapy who were admitted at Vali-Asr and Akbar-Abadi teaching hospitals of the Tehran University of Medical Sciences, Tehran, Iran, from August 2009 through February 2014. Inclusion criteria were: neonates born at ≥ 35 weeks of gestation, exposure to addictive drugs in utero (determined by maternal history and confirmed by positive maternal urine toxicology screens during the last trimester of pregnancy), demonstration of signs and symptoms of NAS requiring treatment (three consecutive Finnegan score of eight or more or two consecutive Finnegan score of 12 or more as calculated every 4 h by the physician). Exclusion criteria were: neonates born at < 35 weeks of gestation, neonates who had intra uterine growth retardation (IUGR), neonates with congenital anomalies, neonates with major concomitant medical illness requiring oxygen therapy, intravenous fluids or medications, neonates with neurologic abnormality, breastfed neonates, concomitant maternal Benzodiazepine or alcohol use, neonates whose parents refused to give informed consent.

**Fig. 1:**
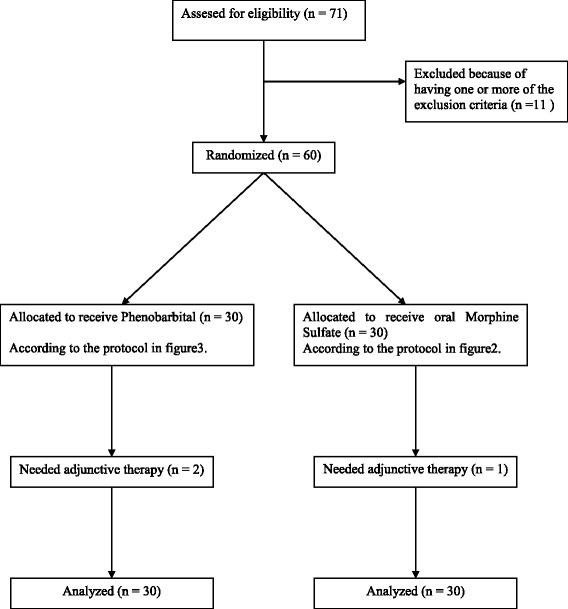
Flow Chart of the study showing patients randomization

After explaining the whole procedure an informed written consent was obtained from the parents, the neonates then were randomly allocated in two groups: the oral Morphine Sulfate treated group and the Phenobarbital treated group (Fig. 1).

A computerized random number generator was used for sequence generation which was carried out by M.S. Simple randomization with a 1:1 allocation ratio was used in this study. We used the consecutive opaque envelopes for the allocation concealment which was performed by F.N. The envelopes were opaque when held to the light, and opened sequentially and only after the participant’s name and other details were written on the appropriate envelope. The implementation of assignments was carried out by H.D.

The primary outcome of our study was the duration of pharmacologic treatment needed for the resolution of symptoms of NAS. Secondary outcomes were the total duration of hospital stay, and also the requirement of adjunctive therapy in each group.

This study was approved by the Research Deputy and the Ethics Committee of the Tehran University of Medical Sciences. (Reference ID = 88-03-30-9106).

This study is registered at the Iranian Registry of Clinical Trials (www.irct.ir) which is a Primary Registry in the WHO Registry Network. (Registration Number: IRCT201406239568N8)

### The protocol

In the randomly assigned oral morphine treatment group, initially 1 ml of a 10 mg Morphine Sulfate vial was diluted using 24 ml of distilled water, to make final concentration of 0.4 mg/ml of Morphine, then depending on the Finnegan score drug was administered in 6 divided doses/24 h as follow: for a Finnegan score of 8–10: 0.24 mg/kg/24 h (0.6 ml/kg/24 h), for a Finnegan score of 11–13: 0.48 mg/kg/24 h (1.2 ml/kg/24 h), for a Finnegan score of 14–16: 0.64 mg/kg/24 h (1.6 ml/kg/24 h), and for a Finnegan score of 17 and above: 0.8 mg/kg/24 h (2 ml/kg/24 h) of Morphine Sulfate was administered. These doses of Morphine Sulfate continued until Finnegan score remained below 8 for 72 h then the drug was weaned gradually by 10 % of maximal dose every day until it was entirely discontinued at the cessation dose of 0.1 mg/kg/24 h of Morphine Sulfate (Table 1).

**Table 1:** The protocol of treatment with oral morphine sulfate

Finnegan score	Volume of 0.4 mg/ml diluted morphine sulfate	Dosage of morphine sulfate
	(ml/kg/24 h)	(mg/kg/24 h)
8-10	0.6	0.24
11-13	1.2	0.48
14-16	1.6	0.64
17 and above	2	0.8
Continued until Finnegan score remained below 8 for 72 h then ⇩ The drug was weaned gradually by 10 % of maximal dose every day until it was entirely discontinued		
Cessation dose: less than 0.1 mg/kg/24 h		

In the randomly assigned Phenobarbital treatment group, initially a loading dose of 20 mg/kg IV or IM was administered then if the symptoms persisted another 5 mg/kg was given every 8–12 h until the entire loading dose would reach a maximum of 40 mg/kg. The maintenance dose was adjusted based on the loading dose and was initiated 12–24 h after the last loading dose as follow: for a loading dose of 20 mg/kg the maintenance dose was 5 mg/kg/24 h, for a loading dose of 30 mg/kg the maintenance dose was 6 mg/kg/24 h, and for a loading dose of 40 mg/kg the maintenance dose was 8 mg/kg/24 h administered IM or IV. These doses of Phenobarbital continued until Finnegan score remained below 8 for 72 h then the drug was weaned gradually by 10 % of maximal dose every day until it was entirely discontinued (Table 2).

**Table 2:** The protocol of treatment with phenobarbital

Initial loading dose of 20 mg/kg IV or IM ⇩ If the symptoms persisted another 5 mg/kg was given q 8–12 h (the maximum loading dose = 40 mg/kg)	
Loading dose (mg/kg)	Maintenance dose (mg/kg/24 h)
20	5
30	6
40	8
Continued until Finnegan score remained below 8 for 72 h then ⇩ The drug was weaned gradually by 10 % of maximal dose every day until it was entirely discontinued	

### Statistical analysis

Sample size was calculated for a power of 80 %, α = 0.05, β = 20 %, and a standard effect size of 0.84. All the statistical analyses were performed using SPSS statistical software (version 18.0.0: PASW, Chicago, IL). Data were displayed using Mean, Standard Deviation (SD) and Range. Mean comparisons between two groups were performed using the *t*-test for independent samples. Furthermore The Chi-squared analysis, Fisher’s exact test, independent-samples *T* test, One-Way ANOVA were used to examine the relationship between maternal drugs and demographic factors in two groups. The level of statistical significance was set at P value < 0.05.

## Results

### Descriptive statistics

This study evaluated 60 neonates with NAS who had three consecutive Finnegan score of eight or more or two consecutive Finnegan score of 12 or more as calculated every 4 h by the physician. 30 neonates were randomly assigned to receive oral Morphine Sulfate and the other 30 were randomly assigned to receive Phenobarbital.

In the Morphine treated group the mean ± standard deviation (SD) for maternal addiction duration was 4.4 ± 2.3 years; gestational age was 37.6 ± 1.4 weeks; birth weight was 2774 ± 487 g; 5-min Apgar score was 9.5 ± 0.6; Finnegan score before treatment was 11 ± 1.8; neonatal age before treatment was 39.7 ± 2.5 h. 16 neonates were female (53.3 %) while 14 neonates (46.7 %) were male.

In the Phenobarbital treated group the mean ± standard deviation (SD) for maternal addiction duration was 3.8 ± 3 years; gestational age was 37.6 ± 1.8 weeks; birth weight was 2726 ± 490 g; 5-min Apgar score was 9 ± 1; Finnegan score before treatment was 11.8 ± 2.2; neonatal age before treatment was 34.6 ± 2.5 h. 17 neonates were female (56.7 %) while 13 neonates (43.3 %) were male.

No significant differences were observed in the demographics between the two treatment groups (Morphine versus Phenobarbital).

### Drugs abused by mothers

The consumed drugs by mothers were Opium, Heroin, Methadone, Cocaine, and Methamphetamine. No significant differences in the maternal consumed drugs were observed between the two treatment groups (Morphine versus Phenobarbital) (Table 3).

**Table 3:** Drugs abused by mothers of the neonates randomized to Morphine versus Phenobarbital treatment group

Drug	Morphine group	Phenobarbital group	*P* value
	N (%)	N (%)	
Opium	12 (40 %)	7 (23.3 %)	0.13 (N.S)
Heroin	2 (6.6 %)	1 (3.3 %)	0.55 (N.S)
Methadone	2 (6.7 %)	2 (6.7 %)	1 (N.S)
Cocaine	6 (20 %)	9 (30 %)	0.37 (N.S)
Methamphetamine	1 (3.3 %)	1 (3.3 %)	1 (N.S)
Polydrug^a^	7 (23.3 %)	10 (33.3 %)	0.28 (N.S)

^a^A combination of the above drugs, N.S: Non Significant

### Effect of treatment allocation on the study outcomes

**Fig. 2:**
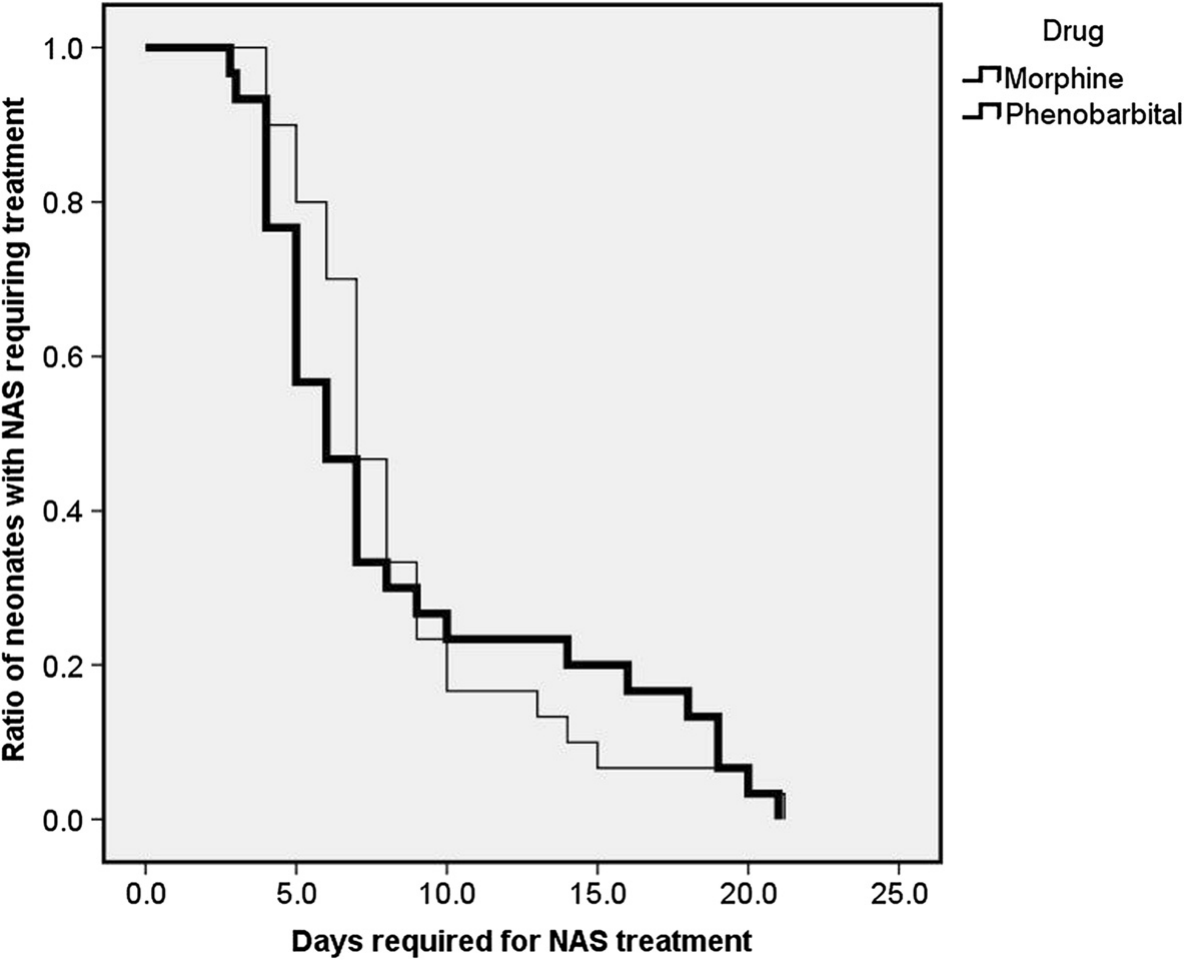
Kaplan-Meier curve showing the effect of Morphine (thin line) versus Phenobarbital (thick line) treatment on the duration of treatment required for the resolution of NAS

The primary outcome of our study was to measure the duration of pharmacologic treatment required for the resolution of NAS symptoms. The mean ± SD for the duration of therapy in the oral Morphine Sulfate group was 206.3 ± 120 h (8.5 ± 5 days) with a range of 68–504 h (2.8–21 days) while in the Phenobarbital group was 204 ± 97 h (8.5 ± 4 days) with a range of 96–510 h (4–21.2 days). There was no significant difference in the duration of pharmacologic therapy required for NAS resolution between Morphine Sulfate and Phenobarbital treated groups (Table 4 and Fig. 2).

**Table 4:** Comparison of the treatment duration, hospital stay and need to adjunctive therapy in the morphine sulfate versus phenobarbital treated groups

Outcome	Morphine sulfate group	Phenobarbital group	*P* value
Duration of treatment	8.5 ± 5 (days)	8.5 ± 4 (days)	0.9
(Mean ± SD)	206.3 ± 120 (hours)	204 ± 97 (hours)	(N.S)
Duration of H.S	12.6 ± 5.6 (days)	12.5 ± 5.3 (days)	0.7
(Mean ± SD)	304 ± 136 (hours)	300.5 ± 128 (hours)	(N.S)
Needing Adjunctive Tx	1 (3.3 %)	2 (6.6 %)	0.5
N (%)			(N.S)

S.D: standard deviation, H.S: hospital stay, Tx: therapy, N.S: Non Significant

One of the secondary outcomes was the total duration of hospital stay in each group; the mean ± SD for the duration of total hospital stay in the oral Morphine group was 304 ± 136 h (12.6 ± 5.6 days) while in the Phenobarbital treated group was 300.5 ± 128 h (12.5 ± 5.3 days). There was no significant difference in the duration of total hospital stay between Morphine Sulfate and Phenobarbital treated groups (Table 4).

Another secondary outcome was the requirement for an adjunctive treatment to suppress NAS symptoms; one neonate in the oral Morphine Sulfate group (3.3 %) and 2 neonates in the Phenobarbital treated group (6.6 %) did not show good therapeutic response and needed an adjunctive therapy. In the Morphine Sulfate group Phenobarbital, and in the Phenobarbital group Morphine Sulfate were used as adjunctive treatments. The need for adjunctive therapy failed to show statistically significant difference between the two groups (Table 4).

## Discussion

In this randomized clinical trial we evaluated 60 neonates with NAS who were born to illicit drugs dependent mothers, there were no significant differences in the duration of treatment required for the resolution of NAS, duration of total hospital stay, and the requirement for adjunctive treatment, between the neonates randomized to receive oral morphine sulfate and the neonates randomized to receive Phenobarbital. Currently the American Academy of Pediatrics (AAP) recommends treating opioid withdrawal with an opioid, while Phenobarbital is only used as the adjunctive treatment for opioid withdrawal in the United States. The results of this paper show that using loading with titration doses of Phenobarbital can be also a good and reasonable first line option especially for the neonates with NAS who were exposed to polysubstance in utero, however more studies with larger sample sizes are needed to confirm these results.

Morphine Sulfate is the most commonly used drug as the first line treatment of both opioid and polydrug withdrawal [18]. Many protocols for NAS treatment prefer morphine because of its effectiveness, predictable half-life and ease of administration [3]. However the risks of prolonged and increased total cumulative exposure to opioids require attention; the evidence suggest that the opioids might negatively affect the growing brain through a dose dependent way, these effects include the reduction of brain size, weight, proteins, RNA, DNA, and neurotransmitters. These concerns made the researchers search for alternatives to the opioids in the management of NAS [12].

Phenobarbital is the second most commonly used drug for the management of both opioid and polysubstance withdrawal [4, 18]. It is mostly used to treat NAS due to polydrug withdrawal and also neonatal seizures due to drug withdrawal [4, 5, 18]. Phenobarbital has been shown to be superior to other sedatives in the management of NAS [19]. and also it seems to have a particular utility in neonates with polydrug exposure in utero [4, 13]. However, concerns about Phenobarbital use in the literature include impairment of suckling, and adverse effects on the developing brain in long-term therapy [3].

Phenobarbital have been used in different protocols for the treatment of NAS; titration dose alone, loading dose with titration, short course and long course of treatment [19]. in the study of Finnegan et al. the time required to control NAS symptoms was significantly lower when a loading dose of Phenobarbital was used [20]. In another study Kahn et al. compared the short and long courses of Phenobarbital treatment, they reported that there were no significant differences in treatment failure and the requirement for adjunctive therapy in short versus long courses of Phenobarbital treatment [21].

In the current study no significant differences were observed in the efficacy of Morphine Sulfate versus Phenobarbital in the treatment of NAS. This is in contrast to the previous studies; Jackson et al. and Ebner et al. both reported that Morphine sulfate was superior to Phenobarbital in the management of NAS due to shorter duration of therapy [14, 15]. several factors could explain the differences observed in the results compared to the previous studies: 1. In the study of Jackson et al. and Ebner et al. due to blinding no loading dose of Phenobarbital was used, this could have introduced bias in the favor of Morphine Sulfate. 2. The types of drugs used by mothers; all the mothers in the study of Jackson et al. and Ebner et al. had received opioids in their pregnancy, while in our study not all the neonates were exposed to opioids in utero, and also in Jackson’s study 22 % in the Morphine group and 44 % in the Phenobarbital group had used Benzodiazepines while mothers who had used Benzodiazepines were excluded from our study. 3. The scoring system and inclusion criteria: in Jackson’s study Lipsitz scoring system was used to evaluate NAS severity and treatment, in Ebner’s study Finnegan scoring system was used and the neonates with Finnegan score of 10 or more were included in the study, while we used Finnegan scoring system in this study and the neonates with Finnegan score of eight or more were included in the study. In addition in our study breastfed neonates were excluded due to the possible effect of breastfeeding on NAS symptoms while in the previous studies these neonates were not excluded.

The main strength of our study was using the loading and maintenance dose of Phenobarbital and comparing it to the effect of Morphine Sulfate on NAS treatment. Using short course of Phenobarbital treatment, involving mothers with substance abuse other than opioids and polysubstance users in the study were other strengths of the study. There are several limitations in our study that should be mentioned for future research: 1. we didn’t measure the serum level of Phenobarbital in our study, this could help in better and more practical interpretation of the results. 2. measuring the adverse events of the treatments was not among the purposes of the study, comparing the adverse effects could help in better understanding and decision making. 3. In this study women who used nicotine were not excluded of the study, since nicotine has been shown to affect the severity of NAS symptoms, this point should be considered when interpreting the results. 4. Due to the nature of the study, it was un-blinded; this could potentially affect as the results of the study. 5. To minimize the factors that could potentially bias the results of the study, many exclusion criteria were used, therefore the generalizabilty of the findings should be considered when interpreting the results of this study.

## Conclusion

Phenobarbital and Morphine treatments are equally effective in the management of NAS. Phenobarbital treatment when used in a loading followed by maintenance doses can be used as an effective and appropriate initial treatment in the neonates with NAS who were exposed to illicit drugs in utero (opioids, stimulants and polysubstance) and exhibit severe withdrawal signs and symptoms requiring pharmacologic intervention. There were no significant differences in the duration of treatment, duration of hospital stay, and the requirement for adjunctive treatment, between the neonates with NAS who received Morphine Sulfate and those who received Phenobarbital.
